# Our Duty*The following is an extract from an excellent Introductory Address delivered by Dr. Burnett at the London Homœopathic Hospital, October 5th, 1883.

**Published:** 1884-01

**Authors:** J. Compton Burnett

**Affiliations:** Lecturer on Materia Medica to the London Homœpathic Hospital Medical School; London (extract)


### OUR DUTY.*

J. Compton Burnett, M. D., Lecturer on Materia Medica to the London Homœpathic Hospital Medical School.

In medicine, as at present known to the world, the only really catholic practitioner of medicine is the broad-minded scientific homœopath. He alone is not sectarian, but progressive and universal. As we go on, if you will do me the honor of listening to me, I shall hope to show you that we practice homœopathically, not, as our calumniators tell you, because we are narrow sectarians and desirous of holding a distinctive position by ourselves, but because we have gone over the entire field of drug therapeutics, and *tried all* systems and methods. Mark you what I say, because this is very important. We have gone over all systems and methods of the drug treatment of disease; we have studied their various merits and demerits, and this in a genuinely catholic, non-sectarian spirit, and having thus covered the whole ground, we find Homœopathy *the best.* Let your minds dwell upon this point a little, for it alone explains the seeming paradox of our position. At the first blush it seems perfectly obvious that a medical man who adopts a peculiar mode of practice must necessarily be a sectarian. We, as homœopaths, are bitterly reproached with this. Many of the best of the profession say to us, "Drop your name and all will be well, and the breach will be healed. We have no objection to *you*, but to your name." Then why not drop the name? I will tell you. *We cannot drop it, because Homœopathy is practically unknown to the bulk of the profession, and exists as a separate thing.* It is really not we who keep the name alive, but the ignorance of the profession of the subject. When the entire profession advances up to the present standpoint of Homœopathy, then the word medicine will *include* it; and having no separate existence, it could not, in the nature of things, have a name to go by, except as the heading of a chapter in history. What Homœopathy now means is the most advanced point in therapeutics, and this extreme van cannot be given up till the entire profession have reached it. When we say we are homœopaths, we do not mean that there is in medicine nothing else but Homœopathy, but we mean that in the curation of disease by medicines we have found the law of similars our *best* guide. We have arrived at this extreme point, not by

---

* The following is an extract from an excellent Introductory Address delivered by Dr. Burnett at the London Homœopathic Hospital, October 5th, 1883.

springs and bounds, or in a hurry, but after going over all the rest of the field and leaving that as less advantageous. Hence our being homœopaths is *not* the outcome of narrow sectarianism or love of a distinctive name from any motive whatsoever, but the result of a broad, eclectic, catholic survey of the entire field of therapeutics. We do not say there is nothing but our homœopathic advance point; by saying we are homœopaths we indicate our position in the great field of drug therapeutics, and in indicating our own we characterize the position of others. And our characterization signifies that all other modes of using drugs are *far* behind us. We do not say the others have no existence; no, we merely say they are *far* behind us, and hence do not exist for us, just because we have something better—so much better that we wax warm in our zeal, we become enthusiastic, and beckon to our allopathic friends in the *rear* to come on, to press forward to where we *are*. Now, our orthodox friends in the rear have no knowledge of the topography of the region occupied by our army in the van; they remain behind, where we used to be lang syne, and steadfastly refuse to believe we are anywhere at all. We shout back to them that we are in a glorious country with immense resources, and ask them to join us and help us to occupy it and cultivate it for the advantage of humanity, and therefore of us and of them. But they will not believe us. So, remember that if any of you medical students aspire to be in the very van of therapeutic science, you *must* find yourselves with us. You cannot help it. Of course, you may abjure the birthright of a free manhood, and join the crypto-homœopaths. Well, they serve a purpose. So did Judas. And to whom, think you, comes the serene satisfaction of duty done? Not to the crypto-homœopaths, who merely serve as a kind of co-operative asses' bridge; they are what schoolboys call sneaks, and a sneak's reward is theirs. I envy them not. They will do nothing great; they will never feel great, they will never feel *noble*, they will never *be* great; for no sneak ever yet became great. That divine afflatus which makes a noble heart bound on to greatness of aim comes not to the sneaky crypto-homœopath. If we aim high we *may* mount to goodness and greatness of soul and deed, but the sneak is a miserable groveller even when at his highest. Some of you may not share these sentiments. Well, I am content to hold them with the choice few; or, if need be, alone. Now, if the profession at present, for the reasons given, cannot be our judges, and if only medical men can be admitted judges of medical questions, how are the claims of Homœopathy to be settled? How is the world—*i. e.*, our fellow human beings—to know whether our opponents or we are right? The *only* way *at present* open to us is to show that Homœopathy cures better than other systems of drug treatment. Gentlemen, there is *no* other way open to us; either we must be false to therapeutic truth and to our common humanity, or we must follow this course till better times dawn, till the general profession advance to within speaking distance of us. What, do you say you would recommend an appeal *ad populum?* Did you not yourself admit that only medical men can adequately grasp the subject? Yes, I do admit that; but we must do our best, and our best *at present* is to convince the people, and so *compel* the profession to listen to us and give us fair play. But an appeal *ad populum* is beneath our dignity, and is unprofessional. Well, if so, then that dignity is a false sheen and no reality, and the profession is an enemy of mankind. As for me, I will prefer the *mens conscia recti,* and will do my duty. You may hiss these sentiments if you like, but I hold them, and I will express them, and am prepared to stand or fall by them. What! do you tell me that I can hold that our Homœopathy is a great life-saving truth, and yet I dare not proclaim it? What! Do you mean to tell me that Homœopathy cures disease better than any other known mode of drug healing, and yet I must hush it up because an interested, prejudiced editor calls it a "fad"? Do I read that Homœopathy minimizes the hideous ravages of small-pox and robs cholera of its terrors, and yet I may not make it known? It is known to me—thanks to the immortal Hahnemann—thanks also to my honored master and predecessor in this chair, Dr. Hughes—it is known to me that *Aconite* will jugulate a simple fever, and shall I seek to hide this knowledge, of which I and mine have the immense advantage, and thus, hiding knowledge, put myself on the level of a common nostrum-monger? Why should you and I have the boon of such knowledge and not others too? Are we priests of the Dark Ages, that we should band ourselves together to shut up the knowledge of the curative action of drugs, and our mode of finding it out, within our own magic Druidic circle that we call the profession? Do those of you who are such strong professionalists really mean that? If you do, then you are at liberty to burn my doctor's diploma, or throw it into the nearest gutter; for if that is the spirit of the profession of medicine, I would rather be outside it. If that is really the aim of the medical profession, it becomes in the aggregate merely a huge co-operative association of nostrum-sellers; and then to be professional must mean not to impart any knowledge to outsiders, to the end that profits may never grow less.

